# Investigating the molecular relationships of Arabian toads via mitochondrial 16S rRNA gene analysis in Taif city, Saudi Arabia

**DOI:** 10.1371/journal.pone.0322129

**Published:** 2025-06-03

**Authors:** Abdulwahed Fahad Alrefaei, Bandar Hamoud Alosaimi, Raed Hamoud Algethami, Javed Ahmad, Rajesh Jeewon

**Affiliations:** Department of Zoology, College of Science, King Saud University, Riyadh, Saudi Arabia; Arish university, Faculty of agricultural and environmental sciences, EGYPT

## Abstract

Taxonomic studies of the Arabian toad (*Sclerophrys arabica*) are of great interest to better understand phylogenetic relationships and their differences in morphology and distribution. In this study, the genetic diversity and phylogenetic relationships of the *Sclerophrys arabica* are investigated utilizing the mitochondrial 16S ribosomal RNA (rRNA) gene. Tissue samples from 30 individuals were obtained from various places in Taif, Saudi Arabia, and following DNA extraction, PCR and sequencing, phylogenetic analyses were performed. Phylogenetic investigation using Maximum Likelihood (ML) methods found that *S. arabica* belongs to a separate clade within the Bufonidae family, closely related to *Sclerophrys gutturalis*, and exhibits no gene flow with other toad species. *Sclerophrys arabica’s* strong phylogenetic relationships to other *Sclerophrys* species support the genus’ monophyly, but its relatively distant position from other species also reflects evolutionary separations within the Bufonidae family. This research demonstrates the utility of the mitochondrial 16S rRNA gene as a reliable genetic marker in amphibian phylogeny, contributing to our understanding of species relationships and providing a basis for future taxonomic and conservation work.

## 1. Introduction

The Arabian toad (*Sclerophrys arabica*) belongs to the Bufonidae family. This species is endemic to the Arabian Peninsula, with populations in Saudi Arabia, Yemen, Oman and the United Arab Emirates [1]. Saudi Arabia’s distinctive position between temperate and tropical zones gives it a unique climatic and ecological diversity. This geographical location results in a wide range of temperatures and weather patterns, creating varied habitats that support diverse flora and fauna. This fluctuating climate supports diverse habitats such as coastal areas, riverine oases, and wetlands. These environments are home to seven amphibian species, including frogs and toads. Four of these species are indigenous to the Arabian Peninsula: Balletto’s toad (*Sclerophrys tihamica*), Arabian toad (*Bufo arabicus*), Dhofar toad (*Bufo dhurefencis*) and Arabian five-fingered frog (*Euphlyctis ehrenbergii*). The other three species, which are found worldwide, are: Marsh frog (*Pelophylax ridibundus*), European green toad (*Bufo viridis*) and Middle Eastern tree frog (*Hyla savignyi*) [2,3]. It is an opportunistic and mesophilous species that prefers to live in moderately moist environments [3]. The *Sclerophrys arabica* (*S. arabica*) is extremely adaptable and can be found in nearly all environments across the peninsula where water is available [2]. This species has a diverse habitat preference, being found in gardens, gravel areas, wadis and date groves. Its activity pattern is both diurnal and nocturnal, suggesting high adaptability to different environmental conditions. The breeding season is closely linked to the rainy seasons, which is a common trait among many desert and semi-desert species, as water availability significantly impacts reproductive success [1,2,4]. The widespread distribution of *S. arabica* has been observed in various habitats within Saudi Arabia. In southern areas, particularly in the southwestern region of the kingdom, *S. arabica* is notably prevalent [2,5–7]. The International Union for Conservation of Nature (IUCN) has designated all Saudi Arabian amphibian species as Least Concern (LC) [8]. The diversity of amphibian fauna in Saudi Arabia reveals that the family Bufonidae is the most widespread and abundant. Among the seven amphibian species identified, the Arabian toad is the most common [1]. Over the last three decades, numerous changes have occurred in the southern region of Saudi Arabia, including the construction of new roads, the expansion of cities, and the degradation of habitats due to climate change. These developments have significantly impacted the natural habitats of amphibians, particularly wetlands and other environments suitable for their survival [9]. Additionally, many habitats have been adversely affected by the use of pesticides [5].

The 16S ribosomal RNA (16S rRNA) mitochondrial gene is a promising tool for animal species identification due to its speed, precision, cost-effectiveness, and ease of use in PCR amplification. This gene, known for its highly conserved and reliable molecular markers, is predominantly used to reconstruct phylogenetic trees of closely related animals [10,11]. In amphibians, the 16S rRNA gene is regarded as a valuable genetic marker for determining taxonomic affiliations and phylogenetic relationships. Incorporating rRNA sequences into phylogenetic analyses allows researchers to achieve more accurate and detailed representations of the evolutionary tree of life. The 16S rRNA gene strikes a balance between conservation and variability, which facilitates detailed phylogenetic studies. This helps researchers to differentiate evolutionary relationships and classify species with greater accuracy. This gene has been especially beneficial in resolving taxonomic difficulties and providing insights into the evolutionary history of amphibians [12].

Southwest Arabia receives a reasonable quantity of rain, which, together with habitat diversity, contributes to the relative richness of amphibians in the region [3]. In this work, we collected DNA samples from 30 Arabian toads in the Taif district of Mecca province, southwest Saudi Arabia. We amplified the 16S rRNA gene to investigate the genetic diversity of toads and their evolutionary relationships with other toads and frogs. The primary goal is to clarify the toad’s position within the Bufonidae family through mitochondrial 16S rRNA gene sequencing and comparative analysis with related species.

## 2. Materials and methods

### 2.1. Sampling

Tissue samples from 30 Arabian toads were obtained from several sites in Taif, Saudi Arabia. These regions included Basal, Ghadeer AL-Banat, AL-Sail Saghir, Wadi AL-Arrj, AL-Sail, AL-Kabir, Okaz, AL-Hada, AL-Shafa, Widi AL-Qudira and Wadi Jeleel. Table 1 lists the sample details, including the accession numbers. All individual samples were obtained from June-August 2024, utilizing the toe-clipping procedure [13]. All sample collection procedures were executed in strict compliance with the guidelines established by the Research Ethics Sub-Committee (REC) of the College of Sciences at King Saud University (KSU) in Riyadh, Kingdom of Saudi Arabia (KSA) (Ethics Reference No: KSU-SE-22–14). Tissue samples were stored at -20°C for future testing. All collected samples were preserved in ethanol and deposited in the College of Sciences, Zoology Department at King Saud University in Riyadh, Saudi Arabia, for further processing.

**Table 1:** Samples used for mtDNA analysis in this study; species name, location, and GenBank Accession No.

Sample NO.	Species	location	GenBank Accession NO.
1B	Arabian Toad *Sclerophrys arabica*	Basal, Taif	PQ558955
2B	Arabian Toad *Sclerophrys Arabica*	Basal, Taif	PQ558956
3B	Arabian Toad *Sclerophrys arabica*	Basal, Taif	PQ558957
4G	Arabian Toad *Sclerophrys arabica*	Ghadeer AL-Banat, Taif	PQ558958
5G	Arabian Toad *Sclerophrys arabica*	Ghadeer AL-Banat, Taif	PQ558959
6G	Arabian Toad *Sclerophrys arabica*	Ghadeer AL-Banat, Taif	PQ558960
7AL	Arabian Toad *Sclerophrys arabica*	AL-Sail Saghir, Taif	PQ558961
8AL	Arabian Toad *Sclerophrys arabica*	AL-Sail Saghir, Taif	PQ558962
9AL	Arabian Toad *Sclerophrys arabica*	AL-Sail Saghir, Taif	PQ558963
10W	Arabian Toad *Sclerophrys arabica*	Wadi AL-Arrj, Taif	PQ558964
11W	Arabian Toad *Sclerophrys arabica*	Wadi AL-Arrj, Taif	PQ558965
12W	Arabian Toad *Sclerophrys arabica*	Wadi AL-Arrj, Taif	PQ558966
13ALS	Arabian Toad *Sclerophrys arabica*	AL-Sail AL-Kabir, Taif	PQ558967
14ALS	Arabian Toad *Sclerophrys arabica*	AL-Sail AL-Kabir, Taif	PQ558968
15ALS	Arabian Toad *Sclerophrys arabica*	AL-Sail AL-Kabir, Taif	PQ558969
16OK	Arabian Toad *Sclerophrys arabica*	Okaz, Taif	PQ558970
17OK	Arabian Toad *Sclerophrys arabica*	Okaz, Taif	PQ558971
18OK	Arabian Toad *Sclerophrys arabica*	Okaz, Taif	PQ558972
19ALH	Arabian Toad *Sclerophrys arabica*	AL-Hada, Taif	PQ558973
20ALH	Arabian Toad *Sclerophrys arabica*	AL-Hada, Taif	PQ558974
21ALH	Arabian Toad *Sclerophrys arabica*	AL-Hada, Taif	PQ558975
22SH	Arabian Toad *Sclerophrys arabica*	AL-Shafa, Taif	PQ558976
23SH	Arabian Toad *Sclerophrys arabica*	AL-Shafa, Taif	PQ558977
24SH	Arabian Toad *Sclerophrys arabica*	AL-Shafa, Taif	PQ558978
25WQ	Arabian Toad *Sclerophrys arabica*	Widi AL-Qudira, Taif	PQ558979
26WQ	Arabian Toad *Sclerophrys arabica*	Widi AL-Qudira, Taif	PQ558980
27WQ	Arabian Toad *Sclerophrys arabica*	Widi AL-Qudira, Taif	PQ558981
28WJ	Arabian Toad *Sclerophrys arabica*	Wadi Jeleel, Taif	PQ558982
29WJ	Arabian Toad *Sclerophrys arabica*	Wadi Jeleel, Taif	PQ558983
30WJ	Arabian Toad *Sclerophrys arabica*	Wadi Jeleel, Taif	PQ558984

### 2.2. DNA extraction

Tissue samples were sectioned into small fragments, and DNAzol (Invitrogen, UK) was utilized to extract DNA from the tissues, adhering to the manufacturer’s guidelines with minor changes as described by Alrefaei [14]. All DNA extractions were performed in dedicated cleanroom facilities to prevent cross-contamination between samples. Negative controls were included in each extraction batch to detect any potential contamination from reagents or the environment. All tools and surfaces were sterilized with bleach or UV light before use. The tissue samples were homogenized using a handheld glass/Teflon homogenizer. For cell lysis, 40 mg of homogenized tissue was transferred to a 100 µl Eppendorf tube, to which 500 µl of DNAzol was added. The sample was then carefully pipetted up and down to ensure full mixing. The samples were then centrifuged at 9000 rpm for 5 minutes at 4°C. The resultant supernatant was transferred to a fresh Eppendorf tube, 500 µl of 75% ethanol was added, and the mixture was agitated for 10 seconds on a vibrating machine. The mixture was centrifuged at 9000 rpm for 8 minutes at 4°C to precipitate the DNA. The supernatant was removed, and the DNA pellet was washed with 200 µl of 75% ethanol. The samples were centrifuged again for 3 minutes to remove the ethanol. The DNA pellets were allowed to air dry in vertical tubes for 20 minutes. Finally, the DNA was resuspended by adding 100 µl of nuclease-free water and stored at -20°C. The DNA concentration in each sample was determined by measuring the absorbance at 260 nm using spectrophotometry. The extracted DNA was visually validated using UV light after gel electrophoresis on a 1% agarose gel stained with ethidium bromide.

### 2.3. PCR amplification of the 16S rRNA

The 16S rRNA gene was amplified by PCR using the primers 16S (5′-CGCCTGTTTATCAAAACAT-3′) and 16SSH (5′-CCGGTCTGAACTCAGATCACG-3′), following the methodology outlined by Palumbi, with some modifications [15]. To prevent contamination during PCR, we used separate workspaces for DNA preparation, amplification, and post-PCR analysis. DNA templates were handled in a PCR setup area distinct from the post-PCR analysis area, which was equipped with a UV sterilizer to decontaminate surfaces between runs. Negative controls were included in every PCR reaction to ensure that no amplification occurred from contamination. The reaction mixture comprised 8.5 µl of Green Master Mix (2X; Thermo Fisher Scientific, Waltham, MA, USA), 3 µl of each forward and reverse primer (Macrogen, Seoul), 8.5 µl of distilled water, and 1 µl of extracted DNA, resulting in a total volume of 24 µl. A negative control using nuclease-free water was included in every PCR. We used these cycling conditions for PCR amplification: an initial denaturation at 94°C for 4 minutes, followed by 40 cycles of 94°C for 1 minute, 52°C for 1 minute, 72°C for 1 minute, and a final extension at 72°C for 5 minutes. The PCR amplification product was visually verified under UV light using an ethidium bromide-stained 1% agarose gel. Both forward and reverse primers were used to sequence the PCR products at Macrogen, Inc. (Seoul, Republic of Korea).

### 2.4. Sequence analysis and constructing the phylogenetic tree

The sequencing data returned to our lab by the biotechnology company were analyzed. The mitochondrial 16S rRNA gene sequences of *S. arabica* were approximately 493 base pairs long. All of the samples in our research had their 16S rRNA extracted in both directions using PCR. The forward and reverse complements of the reverse primer were aligned with the 16S rRNA sequencing data using the MEGA program. CLUSTAL W was employed for the multiple alignment of the sequences utilizing the default parameters [16]. All sequences collected in this study were uploaded to GenBank under the accession numbers PQ558955-PQ558984. Additional sequences of various amphibian species required for phylogenetic analysis were also retrieved from GenBank. A phylogenetic tree using the software application Molecular Evolutionary Genetics Analysis (MEGA), version XI, was generated to determine the evolutionary links between the Arabian toad and other anuran species [17]. Phylogenetic tree for both the GenBank and study datasets were constructed using Maximum Likelihood (ML) with genetic distance and the Tamura-Nei models. The taxa associated with the bootstrap test (1000 replicates), displayed next to the branches, were determined using Felsenstein’s bootstrap approach [18]. MEGA XI was used as a genetic distance matrix to evaluate evolutionary divergence between sequences [19]. The CLUSTVIS web program online tool was used to design a genetic distance heatmap [20].

## 3. Results

**Fig 1:**
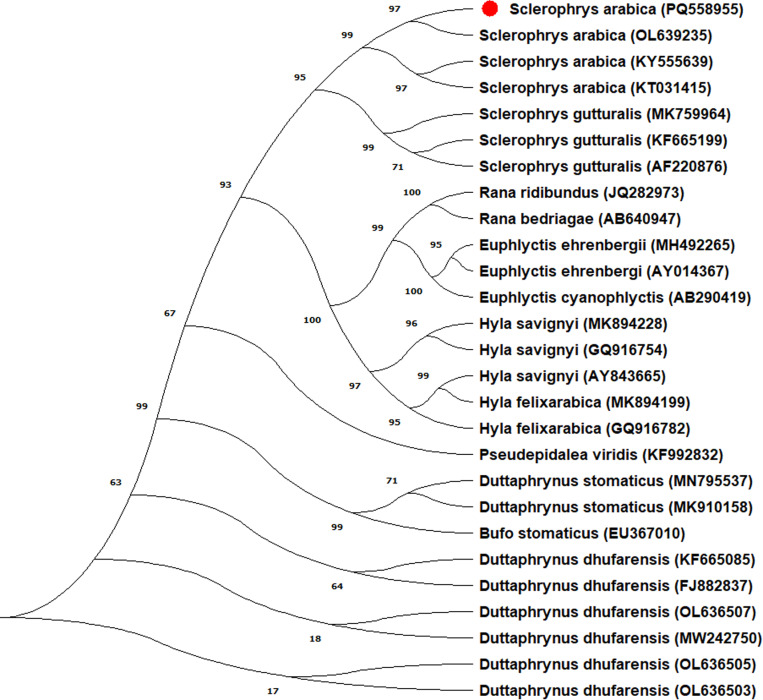
The phylogenetic tree was generated using the Maximum Likelihood method and Tamura-Nei model [21], based on the 16S rRNA gene, indicating the relationships of Arabian toad to other toad and frog species. The percentage of trees that clustered together with the associated taxa are displayed next to the branches. The NCBI GenBank accession numbers for all sequences are included after each species name. This analysis included 27 nucleotide sequences. *Sclerophrys arabica* (PQ558955) is marked in red to emphasize its location in the tree.

**Fig 2:**
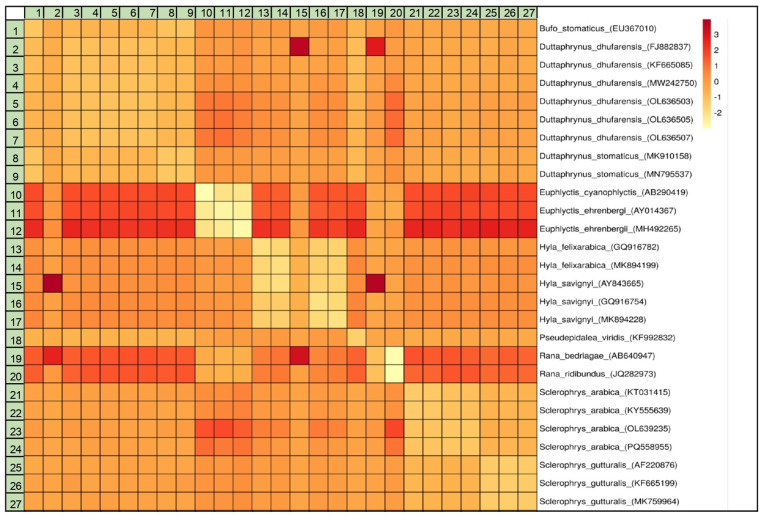
The heatmap shows the relationship or similarity between different species. The colour gradient from red to yellow indicates the intensity or value, with red being higher values and yellow being lower values.

Thirty samples from the Arabian toad were genetically analyzed by 16S rRNA gene amplification and submitted to the GenBank database under the accession numbers PQ558955- PQ558984 (Table 1). All 30 sequences had the same nucleotide structure. The ML tree constructed using the Tamura-Nei model in MEGA XI software with 1000 bootstrap replications and is shown in Fig 1. The tree includes sequences from various species within the genera *Sclerophrys*, *Rana*, *Euphlyctis*, *Hyla*, *Pseudepidalea*, *Duttaphrynus*, and *Bufo*. The ML phylogenetic tree, revealed better insights into the genetic relationships among the species. *Sclerophrys arabica* (PQ558955) clusters with other *S. arabica* sequences (OL639235, KY555639, KT031415) with a high bootstrap value of 97, indicating strong support for this clade. This clade is sister to a group containing *S. gutturalis* sequences (MK759964, KF665199, AF220876), supported by a bootstrap value of 95. The tree indicates that *S. arabica* is more closely related to *S. gutturalis* (Egyptian toad) than to other genera, suggesting a relatively recent common ancestor. *Sclerophrys arabica* (PQ558955) is placed within a clade that includes other sequences of the same species and closely related species. The Genetic Distance Matrix (Table 2 and Fig 2) also supports this position. This positioning indicates that these sequences share a recent common ancestor.

**Table 2:** This table shows the pairwise genetic distance comparisons between different species. It estimates evolutionary divergence between sequences. This analysis involved 27 nucleotide sequences.

Bufo_stomaticus_(EU367010)														
Duttaphrynus_dhufarensis_(FJ882837) 0.04														
Duttaphrynus_dhufarensis_(KF665085 0.04	0.00													
Duttaphrynus_dhufarensis_(MW24275 0.05	0.01 0.01													
Duttaphrynus_dhufarensis_(OL636503 0.06	0.02 0.02	0.01												
Duttaphrynus_dhufarensis_(OL636505 0.06	0.01 0.01	0.00 0.00												
Duttaphrynus_dhufarensis_(OL636507 0.06	0.01 0.01	0.00 0.00 0.00												
Duttaphrynus_stomaticus_(MK910158) 0.01	0.04 0.04	0.04 0.05 0.05 0.05												
Duttaphrynus_stomaticus_(MN795537)0.01	0.04 0.04	0.04 0.05 0.05 0.05	0.00											
Euphlyctis_cyanophlyctis_(AB290419) 0.26	0.25 0.25	0.27 0.31 0.32 0.32	0.26	0.25										
Euphlyctis_ehrenbergi_(AY014367)(2) 0.24	0.23 0.24	0.26 0.30 0.30 0.30	0.25	0.24 0.06										
Euphlyctis_ehrenbergi_(AY014367) 0.24	0.23 0.24	0.26 0.30 0.30 0.30	0.25	0.24 0.06	0.00									
Euphlyctis_ehrenbergii_(MH492265) 0.29	0.28 0.28	0.30 0.32 0.33 0.33	0.28	0.28 0.08	0.03	0.03								
Hyla_felixarabica_(GQ916782)0.15	0.14 0.14	0.16 0.19 0.18 0.18	0.15	0.15 0.24	0.23	0.23	0.27							
Hyla_felixarabica_(MK894199)0.17	0.15 0.15	0.16 0.19 0.18 0.18	0.16	0.16 0.24	0.24	0.24	0.27	0.02						
Hyla_savignyi_(AY843665)0.16	0.32 0.14	0.16 0.19 0.18 0.18	0.15	0.15 0.23	0.22	0.22	0.27	0.02	0.00					
Hyla_savignyi_(GQ916754)0.16	0.15 0.15	0.17 0.20 0.19 0.19	0.16	0.16 0.25	0.25	0.25	0.29	0.05	0.05 0.04					
Hyla_savignyi_(MK894228)0.17	0.16 0.16	0.17 0.20 0.20 0.20	0.16	0.16 0.25	0.25	0.25	0.28	0.04	0.04 0.04	0.02				
Pseudepidalea_viridis_(KF992832) 0.08	0.06 0.06	0.07 0.08 0.08 0.08	0.07	0.07 0.23	0.25	0.25	0.29	0.16	0.17 0.16	0.17 0.18				
Rana_bedriagae_(AB640947)0.22	0.41 0.21	0.25 0.29 0.28 0.28	0.22	0.22 0.23	0.20	0.20	0.25	0.20	0.23 0.34	0.20 0.23	0.23			
Rana_ridibundus_(JQ282973)0.22	0.21 0.21	0.25 0.29 0.29 0.29	0.23	0.23 0.22	0.20	0.20	0.25	0.21	0.22 0.21	0.21 0.23	0.22 0.01			
Sclerophrys_arabica_(KT031415)0.11	0.12 0.12	0.11 0.13 0.13 0.13	0.12	0.12 0.26	0.25	0.25	0.29	0.15	0.16 0.17	0.16 0.17	0.11 0.25 0.22			
Sclerophrys_arabica_(KY555639)0.11	0.10 0.10	0.11 0.13 0.13 0.13	0.10	0.10 0.26	0.26	0.26	0.29	0.15	0.16 0.15	0.17 0.17	0.11 0.23 0.22 0.00			
Sclerophrys_arabica_(OL639235)0.13	0.12 0.12	0.12 0.13 0.12 0.12	0.12	0.12 0.31	0.32	0.32	0.34	0.20	0.19 0.19	0.21 0.20	0.13 0.29 0.29 0.02	0.02		
Sclerophrys_arabica_(PQ558955)0.11	0.10 0.10	0.11 0.13 0.12 0.12	0.09	0.09 0.28	0.27	0.27	0.32	0.16	0.16 0.15	0.18 0.17	0.12 0.25 0.25 0.02	0.02	0.00	
Sclerophrys_gutturalis_(AF220876) 0.09	0.09 0.09	0.10 0.12 0.12 0.12	0.09	0.09 0.25	0.24	0.24	0.30	0.15	0.17 0.16	0.17 0.17	0.11 0.22 0.23 0.07	0.07	0.08	0.07
Sclerophrys_gutturalis_(KF665199) 0.11	0.11 0.11	0.10 0.12 0.12 0.12	0.11	0.11 0.26	0.25	0.25	0.30	0.15	0.17 0.18	0.17 0.17	0.11 0.24 0.23 0.07	0.07	0.08	0.07 0.00
Sclerophrys_gutturalis_(MK759964) 0.10	0.09 0.09	0.11 0.13 0.12 0.12	0.10	0.10 0.25	0.25	0.25	0.30	0.15	0.17 0.16	0.17 0.17	0.11 0.22 0.22 0.07	0.07	0.08	0.07 0.00 0.00

The heatmap (Fig 2) provides a visual representation of the genetic distances among the analyzed species. The colour intensity indicates the degree of genetic divergence, with red representing higher divergence and yellow representing lower divergence. Table 2 displays the genetic distances between different species and sequences. *Sclerophrys arabica* (PQ558955) has small genetic distances from other *S. arabica* sequences. The genetic distance between *S. arabica* and *S. gutturalis* is relatively small (0.12). Genetic distances increase dramatically when compared to more distantly related species, showing the evolutionary difference among amphibian groups. Our analysis confirms that *S. arabica* belongs to a distinct clade within the Bufonidae family. The genetic divergence observed in the 16S rRNA sequences supports the hypothesis that the Arabian toad has undergone significant evolutionary differentiation from its relatives. The clustering of *S. arabica* sequences with high support indicates that these sequences likely represent a monophyletic group within the genus.

## 4. Discussion

The Arabian toad, an amphibian species endemic to the Arabian Peninsula, offers a unique opportunity to study evolutionary relationships within the *Bufonidae* family. Investigating these relationships can yield valuable insights into the biogeographical history and adaptive evolution of this diverse species. Among the various genetic tools available, mtDNA has proven particularly useful in phylogenetic studies. One of the most informative regions of mtDNA is the 16S rRNA gene, known for its conserved nature and variability among different species [7]. The Arabian toad is fascinating from a phylogenetic standpoint, particularly due to its notable morphological variations and unique geographical spread. These characteristics can yield valuable insights into the evolutionary processes that shape adaptation and speciation in amphibians, especially in arid and semi-arid environments like the Arabian Peninsula [22]. The phylogenetic analysis (Fig 1) underscores the distinct genetic identity of *S. arabica* (PQ558955) within the Bufonidae family. The close clustering with other *S. arabica* sequences confirms the monophyly of this species. The separation from *S. gutturalis* suggests an evolutionary divergence that needs further investigation to understand the ecological and geographical factors driving this separation.

Habitat fragmentation often leads to smaller, isolated populations, which can reduce genetic diversity due to increased genetic drift and inbreeding [23,24]. The relatively low genetic distances between *S. arabica* (PQ558955) and *S. gutturalis* (0.12) suggest that these two species share a closer evolutionary relationship than other species in Table 2. This could imply a more recent common ancestor between *S. arabica* and *S. gutturalis*, leading to less genetic divergence. The higher genetic distances between *S. arabica* and species like *Duttaphrynus dhufarensis*, *Euphlyctis cyanophlyctis* and *Rana* indicate greater genetic divergence. These values reflect more distant evolutionary relationships. The low genetic distances between different sequences of *S. arabica* suggest a high level of genetic similarity within this species. This indicates that these sequences are likely from the same or closely related populations, reflecting a lack of significant genetic variation within the species (Table 2). This complication emphasizes the necessity for continued study that employs advanced genomic and morphological analysis to better understand the evolutionary history and diversification of these amphibians. The evolution of Arabian bufonids and their close relatives is a complex biogeographic and taxonomy topic. It is unclear if the unassigned Arabian toads descended from African, Southwest Asian, or Western Eurasian lineages, or if they represent a distinct in-situ diversity on the Arabian Peninsula [22]. This ambiguity highlights the need for comprehensive phylogenetic studies to clarify the origins and evolutionary pathways of these toads, which could provide broader insights into the biogeographical history and evolutionary processes in the region. These results were strengthened by the absence of gene flow among the African and Arabian Peninsula species, which may indicate their different evolutionary lineages.

The mitochondrial 16S rRNA gene is advantageous for examining species, populations, and families due to its reduced mutation rate and lower substitution rates compared to other mitochondrial DNA genes [25,26]. Molecular phylogenetic analyses using markers like RAPD, cyt b, COI, 12S rRNA, and 16S rRNA have significantly advanced our understanding of evolutionary relationships. Each marker has its strengths and limitations, and the choice of marker depends on the specific research question and taxonomic group under study. Combining multiple markers and data types often yields the most reliable and comprehensive results [27–30]. Recently, there has been increased focus on the molecular study of amphibians in Saudi Arabia. One notable study focused on the taxonomic position of the Arabian and Dhofar toad in Wadi Abather, conducted by Alrefaei in 2022. This study contributes to a deeper understanding of the genetic diversity and evolutionary relations among amphibian species in the region [14]. Species from several genera, including *Duttaphrynus*, *Bufo*, and *Sclerophrys*, are included in the evolutionary tree. Other species in the *Sclerophrys* genus, especially *Sclerophrys gutturalis*, formed close groups with *Sclerophrys arabica*. Using mitochondrial 16S rRNA gene sequences, the study discovered that the species sequences created unique clusters with significant bootstrap support, suggesting that *Sclerophrys* has strong evolutionary links. The results indicate that *S. arabica* is closely related to *S. gutturalis*. The lack of gene exchange between African and Arabian Peninsula species suggests that they belong to distinct evolutionary lineages [31].

The clustering of *S. arabica* sequences with high support suggests that they belong to a monophyletic group within the genus. Similar affinities between these species have been shown by earlier evolutionary analyses of the Bufonidae family [14]. These results are supported by the strong bootstrap values, which strengthen the phylogenetic relationship of *Sclerophrys* and related genera. Despite these findings, many aspects of amphibian phylogeny remain unclear, and further research with additional genetic markers is needed to understand the evolutionary history of *S. arabica.*

## 5. Conclusion

The molecular phylogenetic analysis utilizing 16s rRNA indicates that Arabian toad species constitute a distinct clade, whereas other toad species are phylogenetically distant from those of the Arabian Peninsula. This molecular phylogenetic investigation provides valuable insights into the evolutionary relationships of the Arabian toad within the Bufonidae family. The mitochondrial 16S rRNA gene proves to be one of the effective markers for resolving phylogenetic relationships at the species level. Our findings contribute to the broader understanding of amphibian phylogeny and highlight the significance of molecular techniques in evolutionary biology.
